# The cognitive processes underlying affective decision-making predicting adolescent smoking behaviors in a longitudinal study

**DOI:** 10.3389/fpsyg.2013.00685

**Published:** 2013-10-01

**Authors:** Lin Xiao, Gilly Koritzky, C. Anderson Johnson, Antoine Bechara

**Affiliations:** ^1^Department of Psychology, Dana and David Dornsife Cognitive Neuroscience Imaging Center, Brain and Creativity Institute, University of Southern CaliforniaLos Angeles, CA, USA; ^2^School of Community and Global Health, Claremont Graduate UniversityClaremont, CA, USA; ^3^Psychiatry Department and Faculty of Management, McGill UniversityMontreal, QC, Canada; ^4^Department of Neurology, University of Iowa Hospitals and ClinicsIowa City, IA, USA

**Keywords:** adolescents, smoking, decision-making, EV model, Iowa Gambling Task (IGT), longitudinal study

## Abstract

This study investigates the relationship between three different cognitive processes underlying the Iowa Gambling Task (IGT) and adolescent smoking behaviors in a longitudinal study. We conducted a longitudinal study of 181 Chinese adolescents in Chengdu City, China. The participants were followed from 10th to 11th grade. When they were in the 10th grade (Time 1), we tested these adolescents' decision-making using the IGT and working memory capacity using the Self-ordered Pointing Test (SOPT). Self-report questionnaires were used to assess school academic performance and smoking behaviors. The same questionnaires were completed again at the 1-year follow-up (Time 2). The Expectancy-Valence (EV) Model was applied to distill the IGT performance into three different underlying psychological components: (i) a motivational component which indicates the subjective weight the adolescents assign to gains vs. losses; (ii) a learning-rate component which indicates the sensitivity to recent outcomes vs. past experiences; and (iii) a response component which indicates how consistent the adolescents are between learning and responding. The subjective weight to gains vs. losses at Time 1 significantly predicted current smokers and current smoking levels at Time 2, controlling for demographic variables and baseline smoking behaviors. Therefore, by decomposing the IGT into three different psychological components, we found that the motivational process of weight gain vs. losses may serve as a neuropsychological marker to predict adolescent smoking behaviors in a general youth population.

## Introduction

Affective decision-making is one of the most important social functions in our real-life, which enables us to choose wisely according to long-term negative outcomes rather than short-term immediate reward (Bechara, 2005). Impaired affective decision-making has been shown in a variety of neurological and psychiatric conditions such as addiction (Bechara and Damasio, 2002), obsessive-compulsive disorder (Whitney et al., 2004), pathological gambling (Cavedini et al., 2002), and schizophrenia (Sevy et al., 2007; Yip et al., 2009). Recent longitudinal studies also found that affective decision-making capability could predict relapse in addicts (De Wilde et al., 2013) and adolescent binge drinking behaviors (Xiao et al., 2009).

One of the most frequently used neuropsychological tasks to assess affective decision-making in the laboratory is the Iowa Gambling Test (IGT) (Bechara et al., 1994). Compared to other tasks, which assess brain functions related to the calculation of probability or expected value, IGT requires participants to learn and infer from their past experience (such as reward and punishment encountered during the task) about outcome probabilities (Bechara, 2004). Affective and emotional systems therefore play a critical role in such learning processes (Werner et al., 2009; Heilman et al., 2010). The decision-making in the IGT is guided by an emotional signal that assigns negative value for the disadvantageous choices and positive value for advantageous choices, thereby leading behavior toward long term favorable options (Bechara and Damasio, 2005). Recently, IGT or IGT analogous tasks have been used widely from early adolescence to adulthood (Crone and van der Molen, 2004; Hooper et al., 2004; Overman, 2004). Research also found that affective decision-making could be modified by social and environment factors and still develops during adolescence (Xiao et al., 2011).

Recently, researchers found the IGT is a complex task which involves several psychological processes (Busemeyer and Stout, 2002). Using a mathematical model, three different psychological processes can be dissociated from IGT performance: (1) a learning-rate component which indicates the sensitivity to recent outcomes vs. past experiences; (2) a motivational component which indicates the subjective weight the adolescents assign to gains vs. losses (3) a response component which indicates how consistent the adolescents are between learning and responding (Busemeyer and Stout, 2002). The model has been successfully used to discriminate IGT performance among different clinical groups (Stout et al., 2004; Yechiam et al., 2005). Here we apply this approach to study the effects of these psychological processes revealed by the model on the development of adolescent real-life risky behaviors, namely smoking.

Affective decision-making can be affected by other cognitive functions such as working memory (Bechara and Martin, 2004; Toplak et al., 2010). Therefore, in this study, we used the Self-ordered Pointing Test (SOPT) (Peterson et al., 2002) to assess working memory capacity, a task that was developed by Petrides and Milner (1982). This task requires in each trial, an individual to memorize a maximum number of 12 items, either visually or phonologically encoded, and hold them “online” for further operations. Because there are six trials of the SOPT, the maximum capacity is not required in the first trial but the amount of information increases cumulatively over the course of each trial. This process resembles that of transient online storage (Perry et al., 2001), or active monitoring and retrieving of the increasing amount of information (Petrides, 1995) in the concept of working memory. This task has been linked to neural activity within the Dorsolateral Prefrontal Cortex (DLPC) (Petrides et al., 1993) and has been used to assess working memory capacity in several studies (Chey et al., 2002; Pukrop et al., 2003; Chaytor and Schmitter-Edgecombe, 2004; Ward et al., 2005). Moreover, studies have shown that working memory is highly related to general cognitive functions such as reading, mathematics, and reasoning (Engle et al., 1992; Colom et al., 2004; Jarrold and Towse, 2006).

Some previous research studies have found impaired affective decision-making measured by the IGT in adolescent and college smokers (Xiao et al., 2008; Buelow and Suhr, 2012). However, due to the cross-sectional design of these studies, the temporal causal relationship between neuropsychological functions and smoking behaviors remained unclear. That is, these studies could not determine whether abnormalities in their neural systems were the consequence of long-term cigarette use, or whether these abnormalities reflected a developmental predisposition that led to cigarette use. Therefore, the current study tests the ability of psychological processes of affective decision making to account for changes in adolescents' smoking behaviors at a 1 year follow-up. The prospective cohort studies are informative for efficiently investigating a causal relationship between risk factors and adolescent substance use behaviors because it is a longitudinal observation of the individual through time. We also take into account other risk factors reported in previous studies including previous smoking behaviors, working memory capacity, and academic performance. We hypothesize that the psychological processes underlying the IGT at baseline (Time 1) would predict adolescent smoking behaviors 1 year later (Time 2) even after adjusting baseline smoking behaviors, working memory, and academic performance.

## Methods

### Sample

Data collection for this study was supported by the Pacific Rim Transdisciplinary Tobacco and Alcohol Use Research Center, which is investigating social, environmental, and biological determinants of tobacco and alcohol use and abuse among youth in China. All research protocols and instruments were approved by the University of Southern California, Claremont Graduate University, and Chengdu, China CDC Institutional Review Boards. With the assistance of the Municipal Education Committee and the Chengdu Center for Disease Control and Prevention (CCDCP), in Chengdu City, Sichuan Province, four schools were recruited for the study. To ensure maximum variability across the student sample, two academic high schools, one of high- and one of low/middle academic status, and two vocational schools, one of middle- and one of low academic status, were selected. School administrators and teachers from the selected schools agreed to participate in the research after receiving a thorough explanation of the project from the CCDCP staff. One 10th grade class from each of the four schools was randomly selected, and a total of 223 students were invited to participate. Students voluntarily took part in the study and were told that they could discontinue their participation at any time. Out of that total, 16 participants at baseline (Time 1) and twenty-six in the one year follow-up (Time 2) were excluded from the data analysis due to computer malfunctions or failure to complete the survey or follow instructions on the SOPT. The analytic data set included 181 participants (81.2% of total participants) at both the baseline and year 1 study sessions.

### Measures

Baseline (Time 1) measures included two computer-assisted neurocognitive assessments and a paper-and-pencil self-report questionnaire. One year follow-up (Time 2) measures included a paper-and-pencil self-report questionnaire. The instructions for the neuropsychological tasks and the questionnaires were translated into Mandarin Chinese (the only language used in the surveys) and back-translated by the Chinese graduate students in the Pacific Rim Transdisciplinary Tobacco and Alcohol Use Research Center prior to use.

#### Baseline measures

***Iowa gambling task (IGT).***As described in previous studies (Bechara et al., 1994, 1999), the IGT is a computerized version of the gambling task with an automated and computerized method for collecting data. In the IGT, four decks of cards labeled A′, B′, C′, and D′ are displayed on the computer screen. The backs of the cards resemble real decks of cards. The participant starts the task with a sum of make-believe money in his or her account (i.e., $2000), represented by a green bar that changes in length as the participants “wins” or “loses” money during the task. The subject is required to select one card at a time from one of the four decks. When the subject selects a card, a message indicating the amount of money the subject has won or lost is displayed on the screen. The pre-programmed schedules of gain and loss are controlled by the computer. Turning each card can bring an immediate reward of $100 in Decks A′ and B′ and $50 in Decks C′ and D′. As the game progresses, there are also unpredictable losses among the cards. Total losses could amount to $1250 in every 10 cards in Decks A′ and B′ compared to $250 in Decks C′ and D′. Decks A′ and B′ are equivalent in terms of overall potential net losses, and Decks C′ and D′ are equivalent in terms of overall potential net gains over the course of the trials. The difference is that in Decks A′ and C′, the punishments are more frequent but of smaller magnitude. Whereas the punishments in Decks B′ and D′ are less frequent but of greater magnitude. Thus, Decks A′ and B′ are disadvantageous because they yield high immediate gains but greater losses in the long run (i.e., net loss of $250 for every 10 cards), and Decks C′ and D′ are advantageous in that they yield lower immediate gains but smaller losses in the long run (i.e., net gain of $250 for every 10 cards).

The following variables were used for data analysis: (1) After the IGT was completed, a net score was obtained by subtracting the total number of selections from the disadvantageous decks (A′ + B′) from the total number selections from the advantageous decks (C′ + D′). (2) In light of more recent evidence reporting that individuals have a preference for decks with infrequent punishments (Decks B and D) (Overman et al., 2004; Buelow and Suhr, 2012), we calculated scores from the four decks. (3) The IGT net scores chosen in first 40 trials and latter 60 trials were obtained given there is a difference in decision-making between first (decision-making under ambiguity) and latter trials (decision-making under risk) (Brand et al., 2006, 2007; Buelow and Suhr, 2012). (4) parameters of the revised expectancy-valence model over 100 trials were calculated. In the modeling we employed the revised *Expectancy Valence* model (rEV; Busemeyer and Stout, 2002; Yechiam et al., 2005). This is a learning model that predicts the next choice ahead in repeated choice-making. The model has three components, each represented by an estimated parameter.

- Relative weight to gains and losses, measured by the attention-weight parameter. The subjective evaluation of the gains and/or losses obtained upon making a choice is called a valence, and denoted *v*(*t*). It is calculated as a weighted average of the gains and losses resulting from the chosen option in each trial *t*.
  $$v_{j}(t)=w\cdot win(t)-(1-w)\cdot loss(t),$$
  where *win (t)* and *loss (t)* are the amounts of money won or lost on trial *t*; and *w* is the attention weight parameter (0 ≤ *w* ≤ 1).
- Relative sensitivity to recent vs. past outcomes, measured by the recency parameter. The outcomes produced by each alternative *j* are summarized by an expectancy score, denoted *E*_*j*_(*t*), and updated as follows:
  $$E_{j}(t)=E_{j}(t-1)+\phi \cdot [v(t)-E_{j}(t-1)],$$
  where *j* is the selected alternative. The recency parameter, ϕ, describes the degree to which subjective expectancies reflect the influence of the most recent relatively to more distant past experiences (0 ≤ ϕ ≤ 1).
- The effect of expectancies on further choice, measured by the choice consistency parameter. The probability of choosing an alternative is a strength ratio of the expectancy of that alternative, relative to all choice options (using Luce's rule):
  $$\mathrm{Pr}[G_{j}(t+1)]=\frac{e^{\theta (t)\cdot E_{j}(t)}}{\sum_{j}e^{\theta (t)\cdot E_{j}(t)}},$$
  where Pr[G*j*(*t*)] is the probability that alternative *j* will be selected on trial *t*. The term θ (*t*) controls the consistency of the choice probabilities and the expectancies, where: θ (*t*) = *c*^5^ − 1, and *c* is the choice consistency parameter (0 ≤ ϕ ≤ 10).

The accuracy of the model is assessed by comparing its prediction to that of a baseline model. In the baseline model, choices are estimated based on the respondent's mean choices. The estimation procedure is described in detail in Busemeyer and Stout (2002). The statistical test used for comparing the fit of the models was the Bayesian Information Criterion (BIC) for log likelihood differences. Positive values of the BIC statistic indicate that the cognitive model performs better than the baseline model. In the present study, the mean BIC value was 6.24, hence the model fit was adequate.

***Self-ordered pointing test (SOPT).*** We used a computerized version of the SOPT (Peterson et al., 2002), which was based upon a task originally developed by Petrides and Milner (1982). The SOPT has both verbal and non-verbal components with 3 trials of each. In the verbal component, subjects view pictures of concrete, nameable objects (clock, book, bus, etc.); whereas in the non-verbal component, subjects view abstract designs that are difficult to name or encode verbally. In each trial, 12 pages are presented sequentially, with each page depicting the same 12 pictures but in a different spatial arrangement on each page. Subjects are instructed to point to a different picture in each presentation. To effectively select a different picture each time, subjects must retain pictures in working memory. The total number of correct selections of different pictures represents the working memory score. There is a maximum possible score of 12 on each trial and a total of 72 for all 6 trials. In our study, the internal consistency across the 6 trials was 0.86.

#### Questionnaire measurements

***Current smoking.*** Current Smoking status was assessed with this item: “During the past 30 days, have you smoked cigarettes?” Those who indicated smoking in past 30-days were classified as current smokers. Current smoking levels were assessed with this item “During the past 30 days, on the days you smoked, how many cigarettes did you smoke per day? The response options range from “I did not smoke cigarettes during the past 30 days,” “Less than 1 cigarette per day” to “More than 20 cigarettes per day.”

***School academic performance (SAP).*** Students self-reported their academic performance in school by answering the following question: “What is your usual academic performance at your current school or the last school where you received grades?” The five response options ranged from: “Mostly A's, or 90 or more points, or Superior” to “Mostly F's, or Below 60 points, or Failing.” A higher score represented a higher academic performance.

#### One-year follow-up questionnaire measurements

***Measures.*** The same questions in the baseline were used to ask current smoking and current smoking levels. Those who indicated smoking in past 30-days were classified as current smokers.

### Procedures

At baseline (Time 1), trained data collectors from the CCDCP and the University of Southern California provided written and verbal instructions to the students and administered the computer-based assessments and questionnaires in temporary computer labs set up at each school. Students completed the questionnaire and the computer-based assessments (the IGT and SOPT) during a 1 h period. All the students completed the IGT first and then finished the SOPT. Students were provided with earphones to muffle any potentially distracting noises in the environment. One year later (Time 2), students completed the follow-up questionnaire.

### Data analysis

Data were analyzed with the Statistical Package for the Social Sciences for Windows, Version, 17.0 (SPSS Inc., Chicago, IL). Since our sample size (*N* = 181) was relatively large, and since the residuals from the methods satisfied the normality and homoscedasticity assumptions, the variables from EV models, IGT net scores, SOPT, and SAP were treated as continuous without any transformation. The relationships between Time 1 and Time 2 smokers were analyzed using Chi-square tests separately for different levels of current smoking. Independent *t* tests were used to compare measures at Time 1 between current and non-current smokers at Time 1 and Time 2. To reveal potential predictors of current smokers/current smoking levels at Time 2, logistic/linear regression models were used with each of three psychological processes obtained from the IGT performance (Time 1) as the dependent variable and current smokers/current smoking levels (Time 2) as the independent variable, conditioning on Time 1 demographic characteristics, working memory, academic performance, and current smokers/current smoking levels.

## Results

### Relationship between current smokers time 1 and time 2

The relationship between smokers at baseline and year one was shown in Table 1. It shows that 84.5% (*N* = 153) adolescents were non-current smokers at both Time 1 and Time 2. 9.4% (*N* = 17) and 12.2% (*N* = 22) adolescents were current smokers at Time 1 and Time 2, respectively. 6.1% (*N* = 11) adolescents were current smokers at both Time 1 and Time 2. The combination of smokers at baseline was significantly different from that of smokers at year one [χ^2^_(1)_ = 48.53, *P* < 0.001].

**Table 1:** **Relationship between current smokers at Time 1 and Time 2**.

		**Current smokers (Time 1)**		
		**No**	**Yes**	**Total**
**Current smokers (Time 2)**	No	153	6	159
		84.5%	3.3%	87.8%
	Yes	11	11	22
		6.1%	6.1%	12.2%
	Total	164	17	181
		90.6%	9.4%	100%

### Measures among time 1 and time 2 current smokers and non-current smokers

Table 2 shows the measures among Time 1 and Time 2 current smokers and non-current smokers. At Time 1, 88.2% of current smokers but only 46.3% of non-current smokers were males [χ^2^_(1)_ = 10.81, *P* < 0.001]. Moreover, 76.5% of current smokers but only 42.1% of non-current smokers were vocational school students [χ ^2^_(1)_= 7.35, *P* < 0.01]. However, current smokers did not show significant differences with non-current smokers on the measures of IGT net score. Although current smokers chose more from Deck A and B but less from Deck C and D compared to non-current smokers, such differences were not statistically significant for each deck. There were also no differences on three psychological processes underlying decision-making (recency, weight to gain vs. loss, and consistency) or working memory scores. Current smokers scored significantly lower on academic performance than non-current smokers at Time 1 (*P* < 0.05). 41.2% of current smokers at Time 1 reported they have had less than 1 cigarette per day in the past 30 days.

**Table 2:** **Measures in non-current and current smokers**.

**Measures at Time 1**			**Time 1**		**Time 2**	
			**Non-current smokers**	**Current smokers**	**Non-current smokers**	**Current smokers**
Age		(Mean ± *SD*)	16.18 ± 0.55	16.41 ± 0.51	16.18 ± 0.56	16.36 ± 0.49
Gender	Male	N(%)	76 (46.3)	15 (88.2)	73 (45.9)	18 (81.8)
	Female	N(%)	88 (53.7)	2 (11.8)	86 (54.1)	4 (18.2)
School type	Academic	N(%)	95 (57.9)	4 (23.5)	92 (57.9)	7 (31.8)
	Vocational	N(%)	69 (42.1)	13 (76.5)	67 (42.1)	15 (68.2)
IGT net score		(Mean ± *SD*)	4.20 ± 21.52	−2.59 ± 17.20	4.44 ± 19.58	−2.82 ± 28.62
Deck A		(Mean ± *SD*)	19.27 ± 6.22	21.71 ± 5.08	19.53 ± 6.11	20.27 ± 6.06
Deck B		(Mean ± *SD*)	28.63 ± 9.21	29.59 ± 7.70	28.25 ± 8.50	31.14 ± 12.17
Deck C		(Mean ± *SD*)	24.79 ± 7.93	23.65 ± 4.64	24.75 ± 7.07	24.18 ± 11.32
Deck D		(Mean ± *SD*)	27.31 ± 11.32	25.06 ± 10.35	24.47 ± 10.94	24.41 ± 13.05
Recency		(Mean ± *SD*)	0.23 ± 0.36	0.13 ± 0.26	0.23 ± 0.33	0.18 ± 0.31
Weight to gain vs. loss		(Mean ± *SD*)	0.40 ± 0.36	0.37 ± 0.38	0.42 ± 0.37	0.22 ± 0.25^*^
Consistency		(Mean ± *SD*)	3.93 ± 4.01	5.27 ± 4.22	4.11 ± 4.09	3.68 ± 3.73
Working memory		(Mean ± *SD*)	61.81 ± 6.81	60.59 ± 6.60	61.92 ± 6.84	60.27 ± 6.20
Academic performance		(Mean ± *SD*)	3.56 ± 1.07	3.00 ± 1.06^*^	3.56 ± 1.01	3.05 ± 1.07^*^
**DURING THE PAST 30 DAYS, ON THE DAYS YOU SMOKED, HOW MANY CIGARETTE DID YOU SMOKE PER DAY?**							
I did not smoke cigarettes during the past 30 days		N(%)	164 (100)		159 (100)	
Less than 1 cigarette per day		N(%)		7 (41.2)		6 (27.3)
1 cigarette per day		N(%)		3 (17.6)		2 (13.6)
2–5 cigarettes per day		N(%)		5 (29.4)		10 (45.5)
6–10 cigarettes per day		N(%)		2 (11.8)		1 (4.5)
11–20 cigarettes per day		N(%)				1 (4.5)
More than 20 cigarettes per day		N(%)				1 (4.5)

* P < 0.05; Comparing to non-current smokers.

At Time 2, 81.8% of current smokers but only 45.9% of non-current smokers were males [χ^2^_(1)_ = 9.97, *P* < 0.01]. Moreover, 68.2% of current smokers but only 42.1% of non-current smokers were vocational school students [χ^2^_(1)_ = 5.29, *P* < 0.05]. However, current smokers did not show significant differences with non-current smokers on the measures of IGT net score. Although current smokers chose more from Deck A and B but less from Deck C and D compared to non-current smokers, such differences were not statistically significant for each deck. There were also no differences on two psychological processes underlying decision-making (recency, and consistency) or working memory scores. However, current smokers scored significantly lower on weight to gain vs. loss and SAP compared to non-current smokers (*P* < 0.05). 45.5% of current smokers at Time 2 reported they have had 2–5 cigarettes per day in the past 30 days.

### Behavioral performance on the IGT

Previous research showed that the IGT taps into two decision-making contexts, decisions under ambiguity in the first trials and decisions under risk in the latter trials (Brand et al., 2006, 2007; Buelow and Suhr, 2012). We therefore computed the original IGT net score in the first 40 cards selected and last 60 cards selected. For each block, we counted the number of selections from Decks A′ and B′ (disadvantageous) and the number of selections from Decks C′ and D′ (advantageous), and then derived a net score for that block [(C′ + D) - (A′ + B′)]. A net score above zero implied that the participants were selecting cards advantageously, and a net score below zero implied disadvantageous selection.

**Figure 1:**
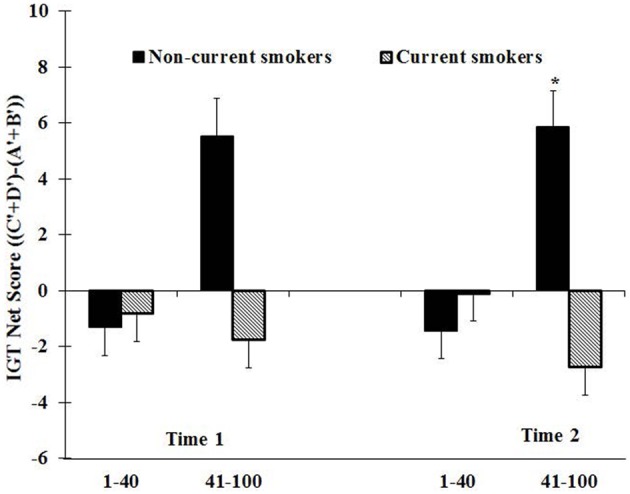
**The IGT net scores [(C′ + D′) – (A′ + B′)] by smoking status (non-current and current smokers) across first (1–40) and latter (41–100) blocks expressed as mean + S.E. after controlling for age, gender, school type, and school academic performance.** Positive net scores reflect advantageous (non-impaired performance) while negative net scores reflect dis advantageous (impaired) performance. ^*^Comparing between groups. ^*^P < 0.05.

Figure 1 presents the net scores as a function of group (non-current smokers and current smokers) and block at Time 1 and Time 2 after controlling for age, gender and school type. At time 1, the IGT performance for smokers is shown on the left panel in Figure 1. The comparison of the plots shows that current smokers at baseline did not differ with the non-current smokers in the first 40 trials on the IGT scores. Although current smokers chose more in disadvantageous decks than non-current smokers, a between-within ANCOVA test did not reveal any significant difference in groups (*non-current smokers vs. current smokers)* or interaction between groups and blocks after controlling for age, gender, and school type (*P* > 0.1).

The IGT performance for smokers at Time 2 is shown on the right panel in Figure 1. The group effect was not significant. However, there was a significant interaction effect between groups and blocks after controlling for age, gender, and school type [*F*_(1, 176)_ = 6.65; *P* < 0.05]. The current smokers did not show difference with non-current smokers in the first 40 trials on the IGT performance. However, they performed significantly worse compared to non-current smokers in the latter trials (*P* < 0.05).

### Variables predicting current smokers at year one

Logistic regressions were performed to predict current smokers at year one at model I in Table 3. The IGT overall net score and three psychological variables were examined individually in four different models after controlling for demographic variables, working memory, academic performance, and baseline current smokers. Among the IGT overall net score and three psychological variables, only weight to gain vs. loss significantly predicted the current smokers at year one after controlling for demographic variables, working memory, academic performance, and baseline current smokers (*P* < 0.05, *OR* = 0.07, 95%*CI* = 0.01, 0.55). Baseline current smoking also significantly predicted the current smoker at year one (*P* < 0.001, *OR* = 21.65, 95%*CI* = 5.17, 90.61).

**Table 3:** **Variables predicting current smokers (model I) and current smoking levels (model II) at Time 2**.

	**B**	**SE**	**Exp (B)**	**95% CI for Exp(B)**	
				**Lower**	**Upper**
**MODEL I**					
Age	0.360	0.539	1.433	0.499	4.122
Gender^a^	−1.126	0.715	0.324	0.080	1.319
School type^b^	0.338	0.751	1.402	0.322	6.110
Working memory	−0.027	0.045	0.973	0.891	1.064
Academic performance	−0.452	0.340	0.636	0.327	1.240
Current smokers (Time 1)	3.075	0.731	21.645^***^	5.171	90.613
Weight to gain vs. loss	−2.673	1.054	0.069^*^	0.009	0.545
**MODEL II**					
Age	0.062	0.088	0.041	−0.111	0.235
Gender^a^	0.117	0.097	0.070	−0.074	0.309
School type^b^	0.168	0.115	0.100	−0.058	0.394
Working memory	0.005	0.008	0.044	−0.010	0.021
Academic performance	−0.056	0.052	−0.071	−0.159	0.048
Current smoking levels (Time 1)	0.750	0.067	0.643^***^	0.618	0.882
Weight to gain vs. loss	−0.280	0.130	−0.122^*^	−0.536	−0.024

a Female as reference group;

b Academic School as reference group.

*** P < 0.001;

* P < 0.05.

Linear regressions were performed to predict current smoking levels at year one at model II in Table 3. The IGT overall net score and three psychological variables were examined individually in four different models after controlling for demographic variables, working memory, academic performance, and baseline current smoking levels. Among the IGT overall net score and three psychological variables, only weight to gain vs. loss significantly predicted the current smoking levels at year one after controlling for demographic variables, working memory, academic performance, and baseline current smokers (*P* < 0.05, Beta = −0.122, 95%*CI* = −0.536, −0.024). Baseline current smoking levels also significantly predicted the current smoking levels at year one (*P* < 0.001, Beta = 0.643, 95%*CI* = 0.618, 0.002). Only the results of the model including the weight to gain vs. loss are presented in Table 3.

## Discussion

We investigated the potential contribution of three different psychological processes (recency, weight to gain vs. loss, consistency) to affective decision-making as measured by the IGT in Chinese adolescents, and their relationship to real-life risky behaviors, namely their smoking behavior, using a longitudinal study design. We found that only weight to gain vs. loss significantly predicted the current smoking behavior one year later. To our knowledge, this is one of the first longitudinal studies to investigate the different psychological processes underlying affective decision-making measured by the IGT in the development of smoking behaviors among adolescents.

Previous studies show that individuals have a preference for decks with infrequent punishments (Decks B and D) (Overman et al., 2004; Buelow and Suhr, 2012), we calculated scores from the four decks and found that current smokers chose more from Deck A and B but less from Deck C and D compared to non-current smokers. However, such differences were not statistically significant for each deck. Therefore, our results could not be explained by the preference of the current smokers for the decks with infrequent punishment. In this study and our previous study (Xiao et al., 2008), the current smokers did not differ on the IGT total net scores over 100 trials compared to non-current smokers. However, in this study, we found that the current smokers at year one performed worse on the latter but not the first trials of the IGT than the non-current smokers, which suggest the current smokers showed impaired decision-making capacity, especially the decisions under risk (Brand et al., 2006, 2007; Buelow and Suhr, 2012). Consistent with these findings, by decomposing the IGT into three different psychological components, we also found that the motivational process of weight gain vs. losses but not consistency and recency processes serves as a neuropsychological marker to predict smoking behaviors one year later in the general youth population. These results also suggest that the sub-processes of affective decision-making measured by the IGT may be more sensitive indictors for adolescent risky behaviors than the IGT net score alone.

Our results were consistent with previous studies which revealed that several populations including young polydrug uses, patients with Asperger and individuals with lesions of the right somatosensory and insula cortex showed impaired in the motivational process of weight gain vs. losses as measured by the IGT (Yechiam et al., 2005, 2008; Johnson et al., 2006b). Therefore, the current smokers in this general adolescent population would be similar to these young polydrug users, patients with Asperger, and individuals with lesions of the right somatosensory and insula cortex. As mentioned in Yechiam et al. (2005), the impairment in the motivational process of weight gain vs. losses may represent difficulties in establishing an emotional representation of the different decks in the IGT. Other studies also show that the right somatosensory and insula cortex is critical for mapping somatic states and translating the raw physiological signals into what one subjectively experiences as a feeling toward the pleasures of gain or the pain of loss (Damasio, 1994; Bechara and Damasio, 2005).

It is interesting that we found only weight to gain vs. loss but not consistency and recency processes linked to the development of adolescent smoking behaviors. The different psychological process underlying affective decision-making measured by the IGT may engage different neural systems. Although to our knowledge no functional imaging study has addressed this topic directly to date, one study examined three psychological processes underlying affective decision-making correlated with gray matter volume (GMV) in healthy controls and patients with schizophrenia (Premkumar et al., 2008) and found that in healthy participants, weight to gain vs. loss was associated with frontal, temporal, parietal, and striatal regions GMV. Recency was associated with GMV in temporal regions, and consistency was associated with GMV in the frontal, temporal, posterior cingulate, and occipital regions (Premkumar et al., 2008). Another study also found genetic factors related to dopaminergic and serotoninergic neural transmitter systems linked to the psychological process of weight to gain vs. loss (Sevy et al., 2006). Future functional imaging and other studies are needed to examine the distinct neural or genetic basis for the three psychological processes underlying affective decision-making.

In our study, working memory as measured by the SOPT performance did not show significant difference between current smokers and never smokers. Although current smokers showed lower school academic performance compared to non-current smokers, it did not predict smoking behaviors at Time 2. Considerable evidence showed that the structural maturational brain processes are continuing well through adolescence, especially in regions and systems associated with risk and reward seeking, emotion regulation and response inhibition (Spear, 2000; Fuster, 2002; Paus, 2005). Specifically, among the latest brain regions to mature without reaching adult levels until the 20 s are some portions of PFC including orbitofrontal, ventrolateral, and medial prefrontal regions (Giedd, 2004; Gogtay et al., 2004). However, studies also show that developmental increases in the IGT performance could not be explained by developmental changes in working memory capacity, inductive reasoning, and behavioral inhibition (Crone and van der Molen, 2004; Hooper et al., 2004), suggesting that maturation of the ventromedial prefrontal cortex may be a developmentally distinct process from maturation of other regions of the prefrontal cortex.

One limitation of the current study is reliance on self-reports of cigarette use, raising the question of whether the respondents reported accurately on their smoking behaviors. However, empirical studies have shown that the self-reported data are, by and large, valid across racial, ethnic, and cultural groups (Wallace and Bachman, 1993; Johnston et al., 1994). Another limitation of our study was that the sample sizes of current smokers are relatively small. However, the prevalence of the cigarettes smoked per day during the past 30-days in our sample was very similar to that in other large-scale population studies in the school students in China (Grenard et al., 2006; Johnson et al., 2006a). Although the rate of cigarette use in our study is lower than the overall rate of U.S. sample, it is comparable to that of Asian students reported in the United States both national and regional studies. For example, compared to high school youth of other racial/ethnic groups, Asian American high school students smoke cigarette at the lowest rate. 10% Asian American students smoke of cigarettes compared with 22% of white and 19% of Hispanic high school students (http://www.healthwellnc.com/TUPCHERITAGETOOLKIT/May/1Fact%20Sheets/Legacy%20Asian%20Americans%20and%20Smoking%20Fact%20Sheet.pdf). Although no legal age has been specified for cigarette use in mainland China, environmental circumstances may be more protective of children in China than in Western countries (more time spent in school and the home, less free time with peers, and less pocket money). This might help explain why in China, uptake and progression to regular smoking continues well into middle adulthood, rather than leveling in adolescence as in the west. Furthermore, statistical significance on both models of current smokers and smoking levels indicates that the effects are robust, and population representativeness of the sample, bolstered by inclusion of students from both major types of Chinese high schools, suggests that the findings are widely generalizable to Chinese youth. However, future studies are needed to establish replicability to other cultural/environmental settings.

In summary, by decomposing the IGT into three different psychological components, we found that the motivational process of weight gain vs. losses significantly predicted adolescent smoking behaviors one year later. Thus, distilling complex decision processes into their underlying components can shed light on real-world choices made by adolescents in the general population. As the EV model has mainly been used in the literature for characterizing the cognitive style of clinical or delinquent populations, the present work demonstrates its potential in a new field. These findings also suggest that intervention targeting the adolescent's motivational process—namely, the relative weighting of gain and loss—may help to reduce smoking behaviors at an early stage.

### Conflict of interest statement

The authors declare that the research was conducted in the absence of any commercial or financial relationships that could be construed as a potential conflict of interest.
